# Magnetic resonance imaging quantification of dehydration and rehydration in vocal fold tissue layers

**DOI:** 10.1371/journal.pone.0208763

**Published:** 2018-12-06

**Authors:** Renee E. King, Kevin Steed, Ana E. Rivera, Jonathan J. Wisco, Susan L. Thibeault

**Affiliations:** 1 Division of Otolaryngology, Department of Surgery, University of Wisconsin-Madison, Madison, Wisconsin, United States of America; 2 Department of Communication Sciences and Disorders, University of Wisconsin-Madison, Madison, Wisconsin, United States of America; 3 Department of Physiology and Developmental Biology, Brigham Young University, Provo, Utah, United States of America; 4 School of Medicine, Ponce Health Sciences University, Ponce, Puerto Rico, United States of America; 5 Department of Anatomy and Neurobiology, Boston University School of Medicine, Boston, Massachusetts, United States of America; 6 Department of Neurobiology and Anatomy, University of Utah School of Medicine, Salt Lake City, Utah, United States of America; McLean Hospital, UNITED STATES

## Abstract

Clinicians commonly recommend increased hydration to patients with voice disorders. However, effects on clinical voice outcome measures have been inconsistent. Hydration-induced change within different layers of vocal fold tissue is currently unknown. Magnetic Resonance Imaging (MRI) is a promising method of noninvasively measuring water content in vocal folds. We sought to image and quantify changes in water content within vocal fold mucosa and thyroarytenoid muscle after dehydration and rehydration. Excised porcine larynges were imaged using proton density (PD) weighted MRI (1) at baseline and (2) after immersion in one of five hypertonic, isotonic, or hypotonic solutions or in dry air. Larynges dehydrated in hypertonic solutions or dry air were rehydrated and imaged a third time. Scans revealed fluid-rich vocal fold mucosa that was distinct from muscle at baseline. Baseline normalized signal intensity in mucosa and muscle varied by left vs. right vocal fold (p < 0.01) and by anterior, middle, or posterior location (p < 0.0001). Intensity changes in the middle third of vocal fold mucosa differed by solution after immersion (p < 0.01). Hypertonic solutions dehydrated the middle third of mucosa by over 30% (p < 0.001). No difference from baseline was found in anterior or posterior mucosa or in muscle after immersion. No association was found between intensity change in mucosa and muscle after immersion. After rehydration, intensity did not differ by solution in any tissue, and was not different from baseline, but post-rehydration intensity was correlated with post-immersion intensity in both mucosa and muscle (p < 0.05), suggesting that degree of change in vocal fold water content induced by hypertonic solutions *ex vivo* persists after rehydration. These results indicate that PD-MRI can be used to visualize large mammalian vocal fold tissue layers and to quantify changes in water content within vocal fold mucosa and thyroarytenoid muscle independently.

## Introduction

Voice disorders affect roughly 30% of the population during the lifespan [1]. Voice therapy for the treatment of voice disorders frequently includes patient education regarding vocal hygiene [2]. A common component of vocal hygiene, echoed by speech-language pathologists, physicians, and singing teachers, is the recommendation to increase systemic hydration by increasing dietary water and avoiding caffeine and alcohol. Two literature reviews and a meta-analysis concluded that hydration and vocal function are likely associated, but that evidence is currently limited [3–5]. One systematic review concluded that acoustic measures of voice production improve with fluid ingestion and worsen with water deprivation [6]; however, direction and significance of change in each parameter was inconsistent among the studies cited. Overall, changes in acoustic, aerodynamic, stroboscopic, perceptual, and self-reported voice outcome measures have been inconsistent and transient following changes in systemic hydration [3,5]. Limitations of clinical studies include failure to control baseline medications and hydration status and insufficient and inappropriately timed measures validating hydration levels [3,6]. Long-term effects of commonly recommended quantities of daily water intake such as “eight 8-ounce glasses” and “half the body weight in ounces” have not been tested for effects on voice production or prevention or treatment of voice disorders.

Vocal folds, housed within the larynx, have high water content. Vocal fold mucosa, which comprises epithelium, basement membrane, and lamina propria, is 83% water [7]. Vocal folds oscillate hundreds of times per second during voice production, and their biomechanics are dependent on the composition and organization of the lamina propria extracellular matrix (ECM) [8,9]. Theoretically, increased water in the superficial layer of the lamina propria could reduce viscosity and therefore reduce phonation threshold pressure (PTP) [10], a measure of ease of voice production defined as the lowest subglottic pressure required to initiate and sustain vocal fold oscillation [11]. However, a meta-analysis found that effects of systemic and/or surface hydration interventions on PTP measures were not significant at the 95% confidence level in people with voice disorders or normal voices [5]. The intermediate layer of the lamina propria is rich in hyaluronic acid, a strongly hydrophilic glycosaminoglycan that regulates tissue viscosity and absorbs shock [12–14], as well as elastic fibers, which impart recoil without deformation to tissues through strong hydrophobicity of elastin [15]. Disordered ECM is characteristic of many voice diagnoses [16]. While biomechanical properties of some of its components are water-dependent on a molecular level, there is no evidence that drinking more water restores vocal fold ECM to a healthy state or prevents its degradation. Deep to the vocal fold mucosa is the thyroarytenoid muscle. Water content in the thyroarytenoid has not been directly measured, but is likely 75–80%, consistent with other striated muscle [17,18]. Vocal fold muscle and mucosa are distinct anatomical entities with different effects on voice function [19]. Systemic or superficial hydration-induced change within different layers of vocal fold tissue is unknown.

Magnetic Resonance Imaging (MRI) is a noninvasive modality that is highly sensitive to water and has been used to image vocal fold layers *ex vivo* in human cadavers, dogs, ferrets, and rats [20–24]. Proton-density weighted MRI (PD-MRI) is a protocol that provides high signal intensity in tissues with high water or fat content [25]. In tissues without abundant fat, such as the ocular lens, it is well-accepted that PD-weighted signal is proportional to water content [26]. In voice science, this is an emerging area of study. Recently, PD-MRI was used to quantify the effect of systemic dehydration on water content of rat vocal folds *in vivo* [27]. Water deprivation that resulted in loss of mean 10.89% body weight decreased signal intensity in vocal folds by a mean of 11.38% and in salivary glands by a mean of 10.74%. Since mean PD-weighted signal change in vocal folds approximated weight loss due to water deprivation and since fat is not abundant in vocal folds, PD-MRI signal is likely proportional to water content in vocal folds, as in other tissues in the body. However, tissue layers within the vocal folds were unable to be resolved in this rodent model [27].

*Ex vivo* studies of larger mammalian larynges hold promise for validating imaging parameters that can reveal vocal fold tissue layers for eventual use in clinical research. Porcine vocal folds, in particular, resemble human vocal folds in overall dimensions and tissue composition, including depth of mucosa [28–30]. Water balance in healthy people is well-regulated by the kidneys such that total body water varies by only 1% over a typical 3-day period [31]. Therefore, the method of dehydration in an imaging study must produce small changes in vocal fold water content, and imaging parameters must be sufficiently sensitive to detect these. There are many excised larynx studies of dehydration and rehydration, but few quantify water loss, and those that do report excessively high dehydration levels. For example, removing less than 30% of water from vocal fold mucosa is not possible using a vacuum oven, a common procedure reported in the literature [7,32,33]. However, immersion in 30% sodium chloride (NaCl) produced a smaller mass decrease in vocal fold mucosa than other methods, calculated at roughly 23%, which is still unacceptably high [34]. In contrast, healthy participants in imaging studies of the effect of acute dehydration on the brain do not lose more than 2–3% body weight over 1–2 days [35–38]. Case reports of death in adolescent wrestlers include 7–9% water weight loss over 3–4 days [39], and 10% is the threshold for severe dehydration in children [40]. In *ex vivo* studies, concentration of hypertonic solutions can be controlled to produce smaller changes, and 5% NaCl was sufficient to induce morphological change in rabbit subglottic lamina propria [41]. Since hypertonic solutions decrease transepithelial resistance [42] and water flux continues in vocal fold epithelium *ex vivo* [43], hypertonic solutions are likely to penetrate epithelium and dehydrate vocal fold lamina propria.

The objective of this study was to validate PD-MRI as a tool to image and quantify changes in water content within separate vocal fold tissue layers after dehydration and rehydration in an excised porcine larynx model. We hypothesized that PD-MRI would allow visualization and measurement of water content in vocal fold mucosa and thyroarytenoid muscle of a large mammalian larynx. We further hypothesized that immersing larynges in solutions of varying concentrations of NaCl would induce physiologic levels of water loss in vocal fold tissues, thus facilitating translation of imaging parameters to *in vivo* large animal and eventual clinical studies.

## Materials and methods

This study was exempt from the Institutional Animal Care and Use Committee (IACUC) of the University of Wisconsin-Madison and Brigham Young University, because tissues were obtained from the slaughterhouse after pigs were slaughtered for human consumption.

### Tissue preparation

**Fig 1:**
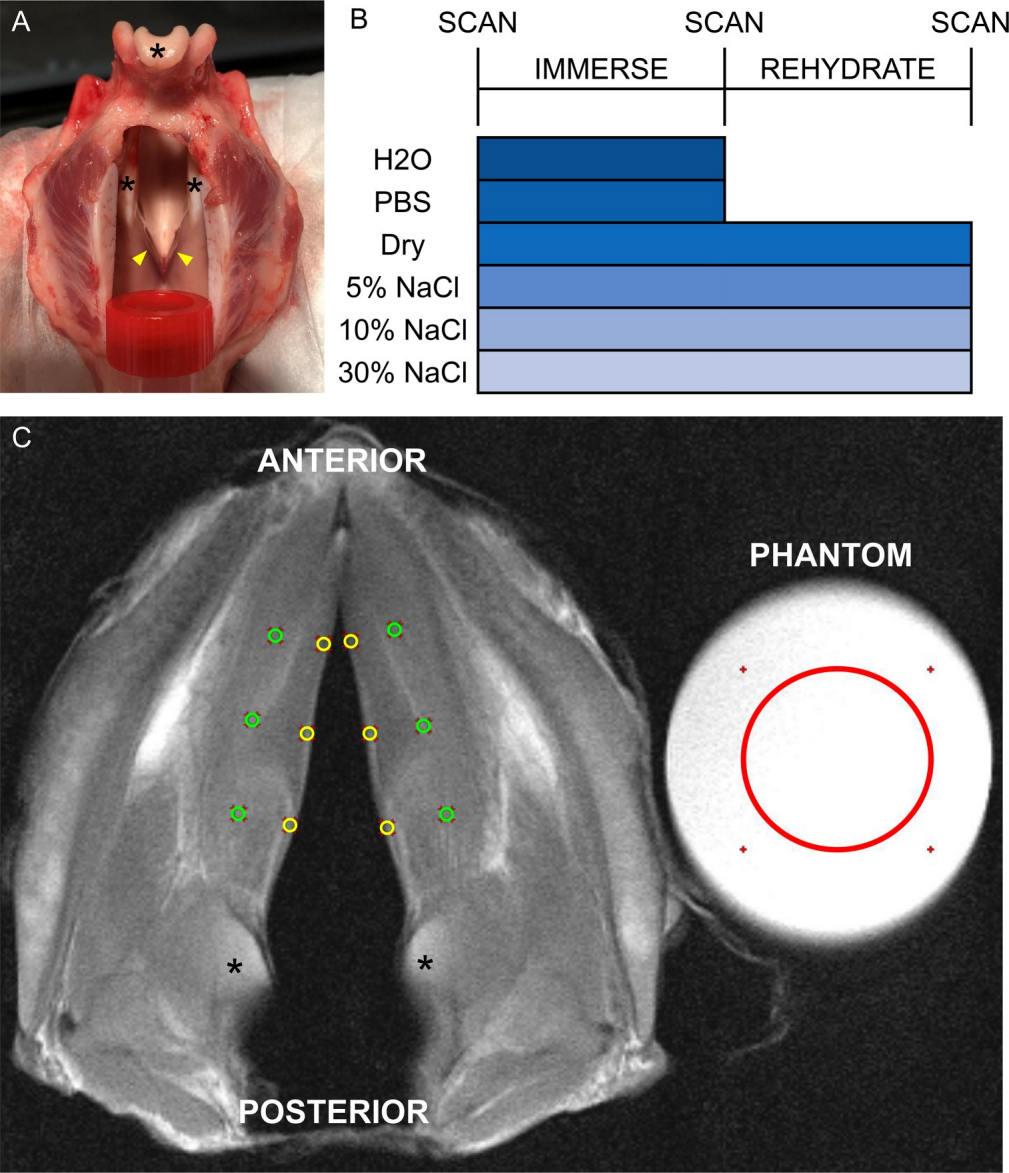
Study procedures. (A): Posterior view of porcine larynx hemisected through the posterior cricoid cartilage and held open with a 15-mL plastic centrifuge tube. Asterisk: arytenoid complex. Arrowheads: membranous vocal folds. (B): Timeline of study procedures. 30 porcine larynges, 5 per group, underwent PD-MRI scans at baseline and after immersion in water (H2O), phosphate-buffered saline (PBS), 5%, 10%, or 30% NaCl, or dry air. Larynges dehydrated in NaCl solutions or dry air were rehydrated in H2O and scanned a third time. (C): Exemplar selections for intensity measurement in a vocal fold slice. Asterisks: arytenoid complex. Yellow circles (D = 0.8–0.9 mm): intensity in anterior, middle, and posterior thirds of membranous vocal fold mucosa. Green circles (D = 0.8–0.9 mm): intensity in anterior, middle, and posterior thirds of thyroarytenoid muscle. Red circle (D = 1.5 cm): intensity of phantom.

A total of 30 porcine larynges were transported immediately postmortem on ice from the slaughterhouse (Circle V Meat Co., Spanish Fork, UT). Larynges were dissected to remove esophagus, excess fat, and strap muscles (omohyoid, thyrohyoid, cricothyroid and sternohyoid) from the thyroid cartilage and the trachea was cut below the second cartilaginous ring. Larynges were inspected to ensure no damage affecting the intrinsic muscles, vocal folds, cricothyroid membrane, or thyroid cartilage in order to prevent basolateral penetration of solution to the vocal folds. To prevent vocal folds from touching and confounding results with capillary action of surface fluid, the posterior cricoid cartilage was hemisected in the superior to inferior direction and held open with a 15-mL plastic centrifuge tube (Fig 1A).

### Immersion and rehydration

For the initial immersion condition, 5 larynges each were immersed in 500 mL of one of five solutions (Fig 1B): hypotonic deionized water (H2O), isotonic phosphate-buffered saline (PBS, pH 7.4, Fisher Scientific, Fair Lawn, NJ, USA), and hypertonic 5%, 10%, and 30% NaCl (Fisher Scientific, Fair Lawn, NJ, USA). Five additional larynges were left dry in a sealed 500-mL container for 30 minutes as controls. We expected that water in vocal fold mucosa would increase after immersion in H2O, remain stable after immersion in PBS, decrease in direct proportion to NaCl content of hypertonic solutions, and slightly decrease in dry air. Larynges dehydrated in hypertonic solutions or dry air were then immersed in 500 mL H2O for 30 minutes to represent rehydration conditions (Fig 1B).

### MRI

A 3 Tesla (3T) TIM-Trio MRI scanner (Siemens Healthineers, Erlangen, Germany) was used for image acquisition. All larynges were imaged at baseline and after immersion. Dehydrated larynges were imaged a third time after rehydration (Fig 1B). Larynges were positioned within a 32-channel head coil with the thyroid prominence superior and the posterior cricoid cartilage inferior. They were wrapped in blue disposable underpads to protect the machinery from the tissues and solutions, then were set on a series of wedges to lift them to isocenter in the coil and stabilized with wedges on top. A vial of H2O was included within the scanner to serve as a phantom for signal normalization. Images were aligned at localization, and 2D axial images of the larynx parallel to the true vocal fold with 4-mm slice thickness were acquired for PD-weighted sequences (echo time (TE) 43 ms, repetition time (TR) 1740 ms) with 6 averages and 2 concatenations giving a scan duration of 14–16 minutes, including localization and scan setup. The sequence field of view was 88mm, base resolution was 384 and slice thickness was 4mm giving a voxel dimension of 0.2mm x 0.2mm x 4mm. Additional parameters include 25% phase oversample, generalized autocalibrating partial parallel acquisition (GRAPPA) turbo factor of 12 and flip angle of 150.

### Image analysis

Digital Imaging and Communications in Medicine (DICOM) files were opened in the RadiANT DICOM Viewer (Medixant, Poznan, Poland). One slice per file was selected just inferior to the vocal process of the arytenoid cartilage to capture the medial surface of the vocal fold. Codes were created with a random number generator (Microsoft Excel, Redmond, WA, USA) and a key was saved in a separate file. Slices were renamed with codes and saved in a separate folder. A total of 80 slices were acquired, 20% of which were saved twice using different codes for determining intrarater reliability, resulting in a total of 96 slices used for measurement.

Coded slices were reopened in RadiANT with annotations off, thus masking group and timepoint during measurement. Measures were taken by a clinician with five years’ experience studying laryngeal anatomy and biology following initial consultation with the senior authors who each have over a decade of experience in medical imaging and porcine vocal fold surgery respectively. Since vocal folds are subject to different forces during vibration along the anterior-posterior axis [44,45], particularly in the “striking zone” of the middle third where phonotraumatic lesions are likely to occur [46,47], vocal folds were measured at multiple locations within mucosa and muscle. Raw signal intensity (arbitrary units) was taken at 13 points per slice: one each in the middle of the anterior, middle, and posterior thirds of left and right vocal fold mucosa and thyroarytenoid muscle, and one in the phantom (Fig 1C). Because porcine vocal fold mucosa is approximately 0.9 mm thick [28,48], mucosa was measured using circular selections with D = 0.8–0.9 mm beginning at the vocal fold edge and moving laterally toward the thyroid cartilage. Muscle was measured using circular selections of the same dimensions placed laterally to the corresponding points in the mucosa. A circle with D = 1.5 cm was used to measure intensity of the phantom. For each slice, normalized intensity for each point within tissue was calculated as a percentage of intensity of the phantom. After normalized intensity was calculated, the key was used to decode data.

### Statistical analysis

Anatomical and group differences in baseline tissue intensity were analyzed using mixed models for mucosa and thyroarytenoid separately with normalized tissue intensity (% of phantom) as the response variable. Fixed effects were side (left or right), location within the vocal fold (anterior, middle, or posterior), and group (H2O, PBS, Dry, 5% NaCl, 10% NaCl, or 30% NaCl). Intercepts for each larynx were included as repeated measures. Tukey’s honestly significant difference (HSD) test was used for post hoc analysis. Post-immersion intensity and post-rehydration intensity were calculated as percentage of baseline, with baseline intensity set at 100%. For analysis of change from baseline, values were averaged between left and right vocal folds for each location within mucosa and muscle. Intensity post-immersion and post-rehydration was analyzed using one-way analysis of variance (ANOVA). Welch’s ANOVA was used for data that failed Levene’s test for equality of variances at α = 0.05. Games-Howell test was used for post hoc pairwise comparisons. Welch’s T-test with Bonferroni correction was used to test the hypothesis that intensity in various subgroups of tissue was not equal to 100%. The Pearson correlation coefficient was used to analyze correlations between mucosa and muscle intensity in immersion and rehydration conditions and correlations between changes in dehydration and rehydration within mucosa and muscle. For correlation analyses of changes in intensity, data were paired by side and location (e.g. dehydrated left anterior mucosa was paired with rehydrated left anterior mucosa and with dehydrated left anterior thyroarytenoid). Intrarater reliability was analyzed using intraclass correlation coefficient (ICC) estimates and 95% confidence intervals (CI) calculated based on a single-rater, absolute-agreement, two-way mixed effects model [49]. Statistical analyses were performed with SAS Studio 3.5 (SAS Institute, Inc., Cary, NC, USA) and RStudio 1.0.143 (RStudio, Inc., Boston, MA USA). The α level for significance was 0.05.

## Results

**Fig 2:**
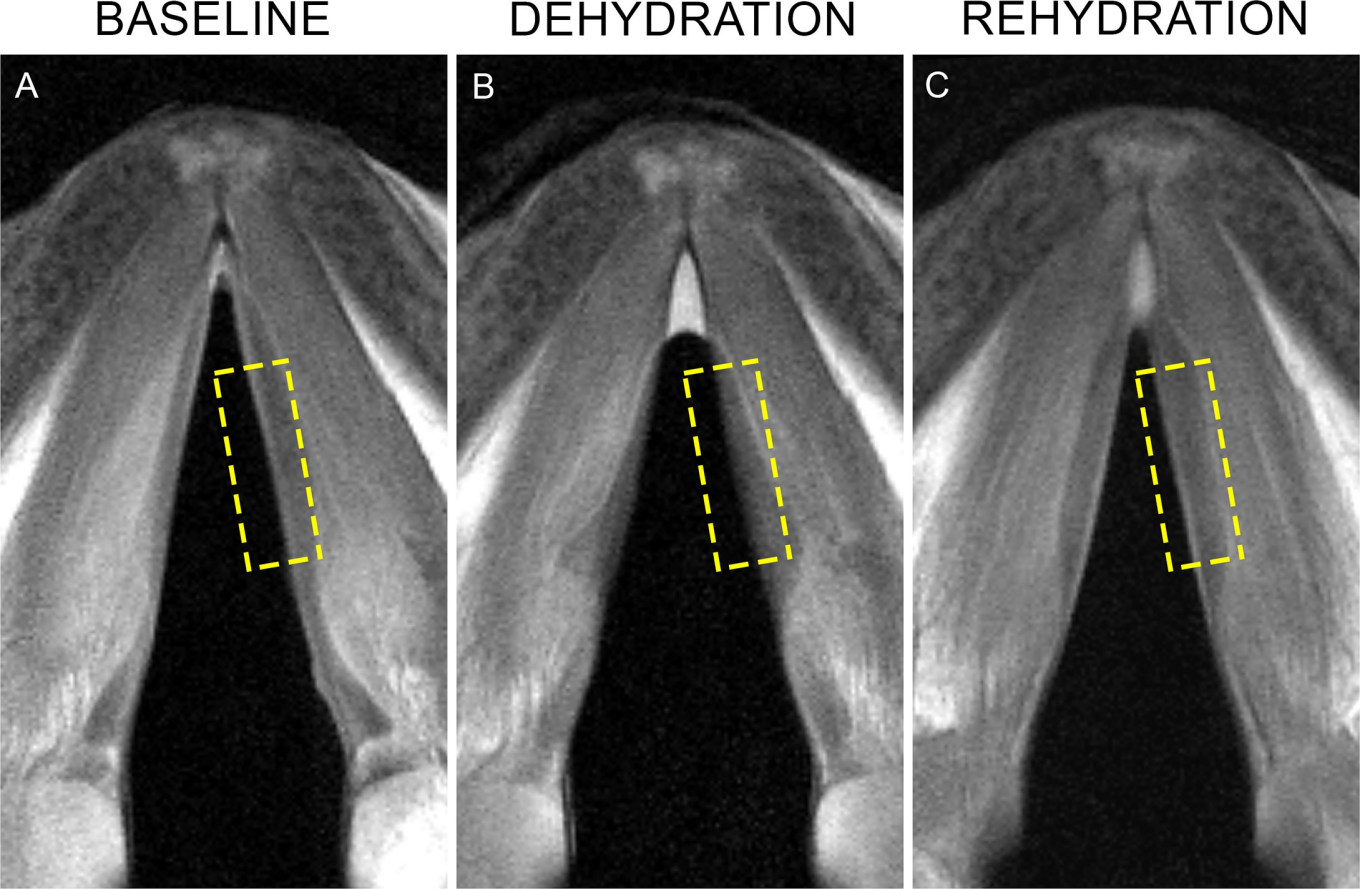
PD-MRI image of porcine vocal fold. Representative images of slices through vocal fold of the same larynx. (A): Baseline. (B): After dehydration in 30% NaCl. (C): After rehydration in H2O. Dashed boxes highlight mucosa at the vocal fold edge.

At baseline, vocal fold appearance in PD-MRI scans was characterized by a bright line at the vocal fold edge, indicative of high fluid content in mucosa (Fig 2A). Muscle and cartilage appeared in shades of gray as expected with PD-MRI. After dehydration in hypertonic solutions, fluid in mucosa was no longer visible (Fig 2B) but was restored after rehydration (Fig 2C). In all scans, the phantom was bright white.

### Intrarater reliability

ICC between first and second raw measurements of tissue intensity (arbitrary units) was 0.9566 with 95% CI = 0.9428–0.9672. ICC between first and second calculated values of normalized tissue intensity (% of phantom) was 0.9533 with 95% CI = 0.9384–0.9646.

### Baseline signal intensity in vocal fold tissue

**Fig 3:**
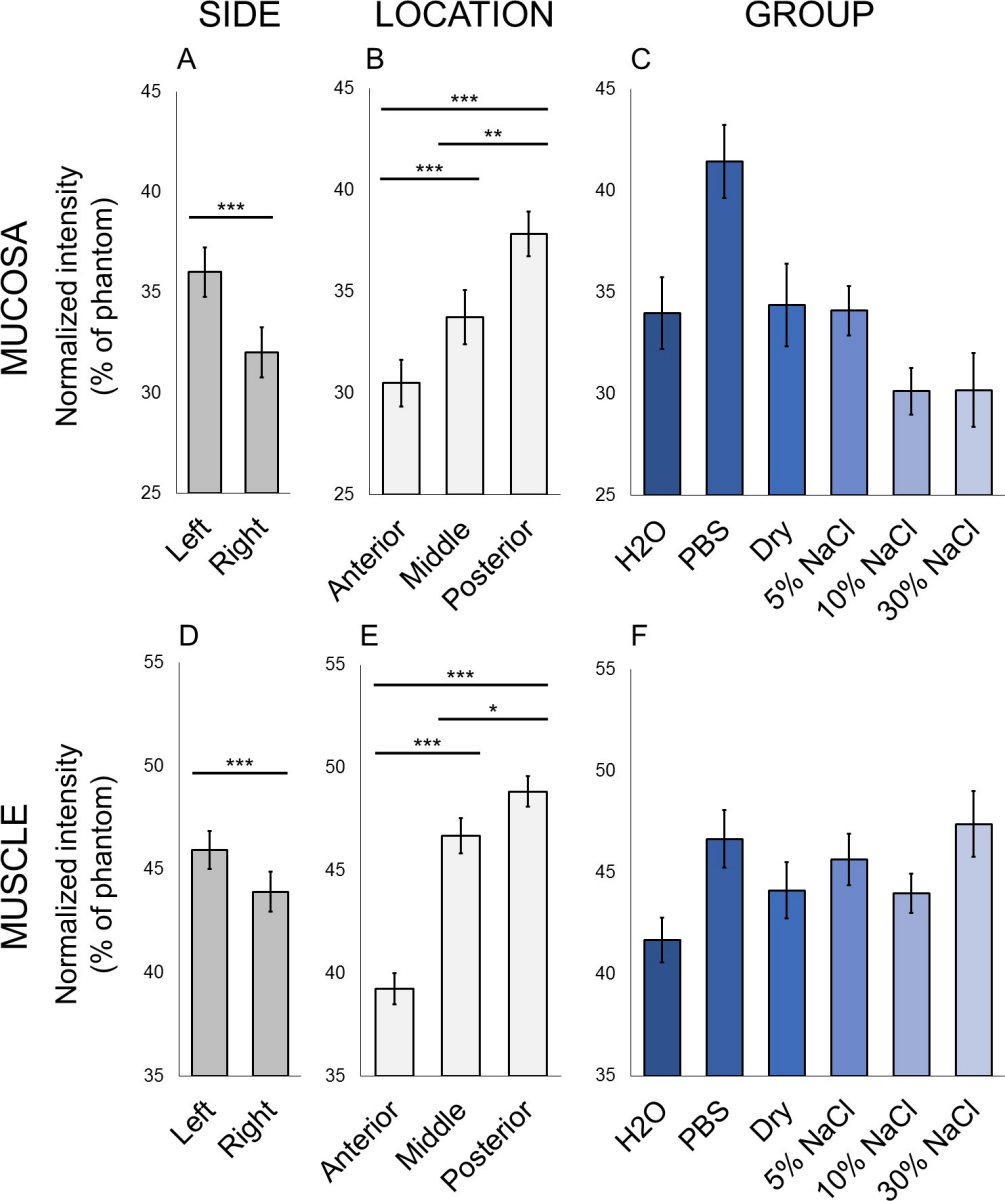
Baseline tissue intensity varied by side and location within vocal folds. Normalized intensity (% of phantom) at baseline in mucosa (A-C) and muscle (D-F). (A) and (D): Left and right vocal fold. (B) and (E): Location in vocal fold. (C) and (F): Experimental group. Data plotted as mean ± SEM. n = 5 larynges per group. *p<0.05, **p<0.01, ***p<0.001: significance of pairwise comparisons of group means using Tukey’s HSD test after significant main effects were found in mixed model ANOVAs.

Baseline intensity, representative of water content [25], was analyzed within mucosa and muscle for differences by side, anterior-posterior location, and group. Within the mucosa, type 3 tests of fixed effects in the mixed model revealed a significant interaction effect of location by group (Table 1). Post hoc testing revealed a significant effect of location within larynges in Dry, 5% NaCl, and 30% NaCl groups, but not those in H2O, PBS, or 10% NaCl groups (Table A in S1 File). There was a significant effect of group within the middle third of the mucosa (Table B in S1 File); however, no pairwise comparisons were significantly different (Table C in S1 File). In both mucosa and muscle, there were significant main effects of side and location on normalized intensity (Table 1). Left vocal fold was brighter than right in both tissue types (Fig 3A and 3D). In both mucosa and muscle, intensity increased from anterior to middle third and from middle to posterior third (Fig 3B and 3E). There was no main effect of group (i.e., planned immersion solution) on normalized intensity of mucosa or muscle (Table 1; Fig 3C and 3F). Taken together, these data indicate that there were no between-group differences in vocal fold intensity at baseline once side and location were accounted for. Therefore, group differences in intensity after immersion and rehydration resulted from experimental conditions.

**Table 1:** Anatomical and group differences in baseline vocal fold tissue intensity.

Effect	Result	Significance (p)
Mucosa		
Side	F(1,24) = 17.95	0.0003***
Location	F(2,96) = 20.25	< .0001***
Group	F(5,24) = 1.97	0.1190
Side × Location	F(2,96) = 0.14	0.8665
Side × Group	F(5,24) = 0.37	0.8666
Location × Group	F(10,96) = 0.33	0.0193*
Side × Location × Group	F(10,96) = 2.28	0.9719
Muscle		
Side	F(1,24) = 9.51	0.0051**
Location	F(2,96) = 78.81	< .0001***
Group	F(5,24) = 1.06	0.4051
Side × Location	F(2,96) = 0.10	0.9082
Side × Group	F(5,24) = 1.53	0.2178
Location × Group	F(10,96) = 1.38	0.1989
Side × Location × Group	F(10,96) = 0.30	0.9786

Type 3 tests of fixed effects on vocal fold tissue intensity in mixed models with intercepts for each larynx included as repeated measures.

*p < .05

**p < .01

***p < .001.

### Immersion

**Fig 4:**
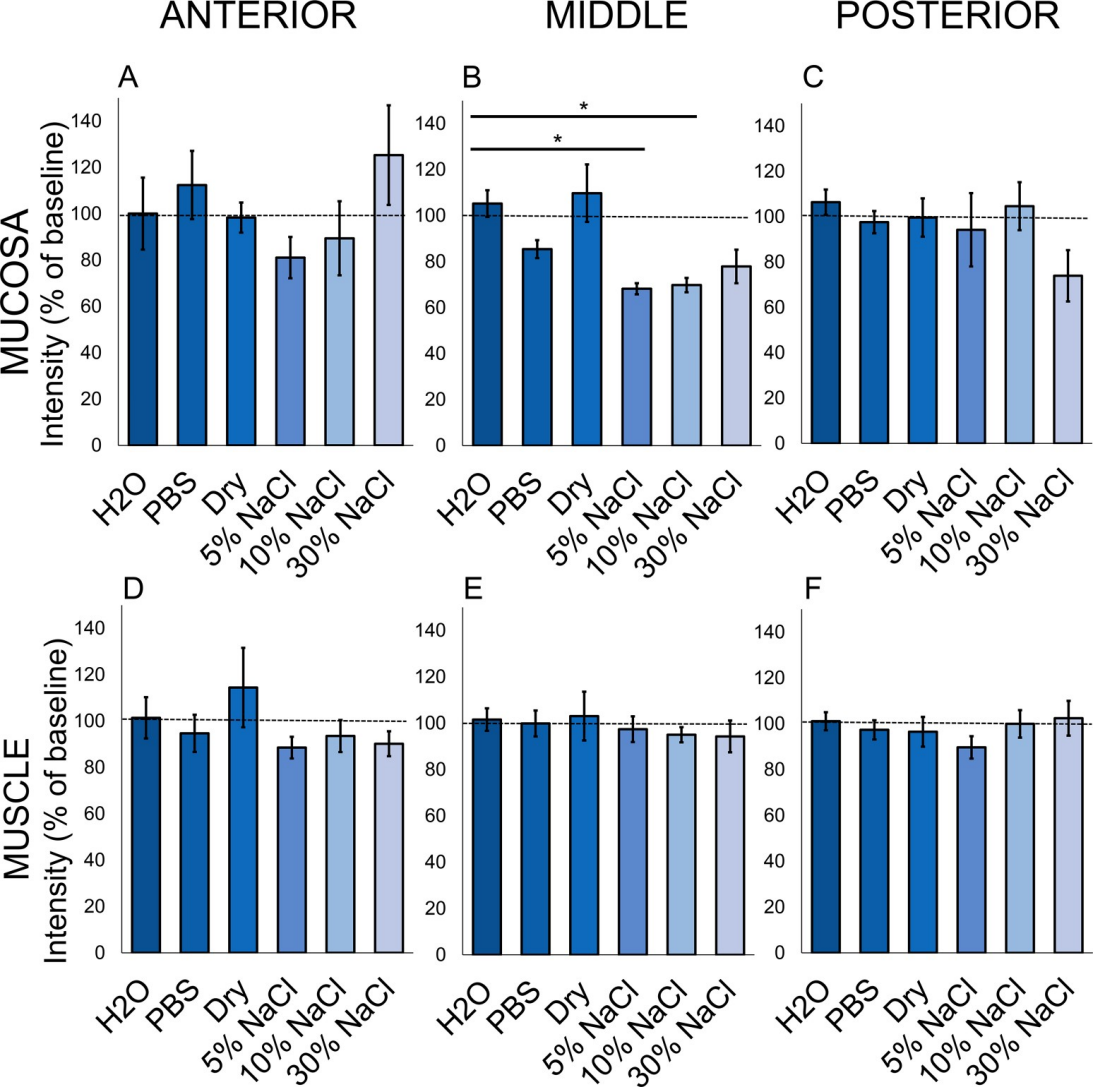
Intensity in vocal fold mucosa and thyroarytenoid after immersion in hypo-, iso-, and hypertonic solutions. Intensity (% of baseline) as a function of immersion solution in mucosa (A-C) and in thyroarytenoid muscle (D-F). Data plotted as mean ± SEM. n = 5 larynges per group. Pairwise comparison using Games-Howell test: *p<0.05.

Post-immersion intensity in the middle third of vocal fold mucosa failed Levene’s test for equality of variances (F(5, 24) = 4.40, p = 0.0055). Welch’s ANOVA revealed significant differences by group after immersion (Fig 4; F(5, 10.8726) = 8.55, p = 0.0017, ω^2^ = 0.55). Midfold mucosa was brighter in larynges immersed in H2O than in those immersed in 5% and 10% NaCl (Fig 4B; adjusted p = 0.011 and 0.012, respectively). Immersion in 5% and 10% NaCl decreased intensity in midfold mucosa (Table 2). There was no evidence that other immersion solutions produced differences from baseline intensity.

**Table 2:** Intensity in middle third of vocal fold mucosa after immersion in hypo-, iso-, and hypertonic solutions.

Group	Intensity (% of baseline)		Significance (p)
	Mean	95% CI	
H2O	105.26	89.22–121.29	0.4142
PBS	85.44	74.67–96.21	0.0199
Dry	109.75	75.05–144.46	0.4789
5% NaCl	68.20	61.51–74.89	0.0002*
10% NaCl	69.81	61.12–78.49	0.0006*
30% NaCl	77.90	57.63–98.16	0.0389

Results of Welch’s T-test of the hypothesis that intensity after immersion was not equal to 100% of baseline. n = 5 larynges per group.

*p < Bonferroni-adjusted α = 0.05/6 = 0.0083.

Post-immersion intensity did not vary by solution in anterior or posterior mucosa (Fig 4A and 4C; Table A in S2 File) and did not vary from baseline intensity (Table B in S2 File). There was no evidence for differences in intensity change by solution in any location in thyroarytenoid muscle (Fig 4D and 4F; Table A in S2 File), or for changes from baseline intensity (Table B in S2 File). Taken together, our data indicate that brief immersion in hypertonic solutions can induce water loss in vocal fold mucosa at the midfold, but not in thyroarytenoid muscle.

### Rehydration

After rehydration, there were no group differences in intensity of any location in vocal fold mucosa or posterior thyroarytenoid by one-way ANOVA (Table A and Fig in S3 File). Anterior and middle thyroarytenoid failed Levene’s test for equality of variances (F(3,16) = 5.19 and 3.37, p = 0.0108 and 0.0445). Welch’s ANOVA revealed no differences in post-rehydration intensity by group in anterior or middle thyroarytenoid (Table A in S3 File). There was no evidence that intensity after rehydration was different from baseline in any group (Table B in S3 File). Therefore, rehydration restored intensity close to baseline levels.

### Correlations

**Fig 5:**
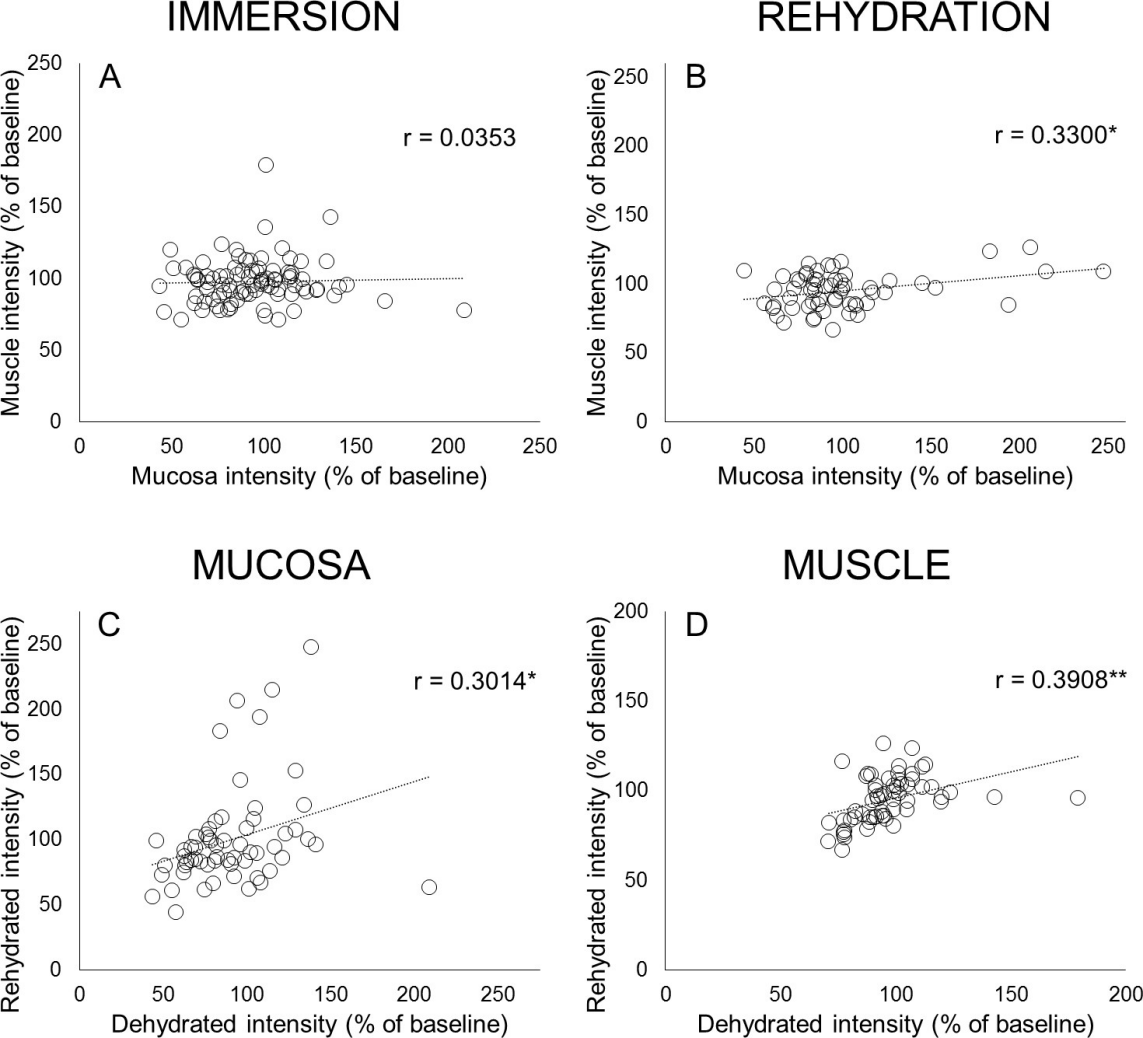
Correlations between intensity changes between tissues and across conditions. (A-B): Correlation between intensity (% of baseline) in mucosa and thyroarytenoid muscle after immersion (A) and after rehydration (B). (C-D): Correlation between intensity (% of baseline) after dehydration and after rehydration in mucosa (C) and muscle (D). Pearson correlation coefficient: *p < .05, **p<0.01.

Correlation coefficient between mucosa and muscle intensity after immersion was not significant (r = 0.0353, p = 0.7412, Fig 5A), indicating that various solutions produced different effects in separate tissue layers. In dehydrated larynges, the correlation coefficient between rehydrated mucosa and muscle was 0.3300 (p = 0.0100, Fig 5B). Correlation coefficient between dehydrated and rehydrated intensity was 0.3014 (p = 0.0193) in mucosa and 0.3908 (p = 0.0020) in muscle (Fig 5C and 5D), implying that despite intensity approaching baseline after rehydration, some effects of dehydration persisted.

## Discussion

This is the first study to our knowledge to expose vocal fold tissue layers on MRI scans without contrast and with clinically feasible acquisition times. Herrera et al. [20] were able to distinguish vocal fold epithelium, lamina propria, muscle, and cartilage, as well as scar and implanted biomaterials, using 11.7T scans of paraformaldehyde-fixed dog and ferret larynges. However, stated limitations included scanning duration that was too long for clinical application. Kishimoto et al. [21] resolved mucosa, muscle, cartilage, and scar tissue in excised rat larynges in a 10-minute scan at 9.4T, but tissues were immersed for 10 days in gadolinium contrast. *In vivo* scans in the same study did not reveal vocal fold tissue layers at 9.4T, even with IV contrast. Chen et al. [22], Klepacek et al. [23], and Wu and Zhang [24] each distinguished mucosa from muscle in human cadaveric larynges without contrast; however, field strength ranged from 4.7-7T and scan times ranged from 2–18 hours. Oleson et al. [27] used a 7T system to demonstrate pre-post changes in rat vocal fold and salivary gland signal intensity after water deprivation, but again could not distinguish vocal fold tissue layers. The scans in the present study were acquired with a 3T system that is widely clinically available and acquisition times of only 14–16 minutes. We revealed a bright, fluid-rich line at the vocal fold edge (Fig 2) consistent with known thickness of porcine vocal fold mucosa that was clearly distinct from muscle.

High variability in intensity within vocal fold tissue at baseline (Fig 3) is consistent with other *ex vivo* studies and hydration literature in general, but could be due to procedural limitations. Specifically, larynges were positioned supine within the scanner with the phantom on the right, so the increase in intensity from anterior to posterior and from right to left may be explained by gravitational flow of water within tissue. Body position affects clinical measures of total body water as well [50]. There was an interaction effect of location by group, with anterior-posterior differences in some groups but not others. There was evidence for group difference in the middle third of the mucosa at baseline, but no pairwise differences were significant and there were no group differences otherwise. By far, the strongest effects on baseline intensity were side, which we accounted for by calculating mean values, and location, which we accounted for by analyzing anterior, middle, and posterior vocal fold locations separately.

We immersed larynges in hypo-, iso- and hypertonic solutions with varying solute concentrations in order to produce changes in vocal fold lamina propria water content through osmosis. Different solutions produced different levels of water content only in the middle third of vocal fold mucosa (Fig 4B). We found that 5% and 10% NaCl dehydrated this tissue 32% and 31% respectively (Table 2), which are non-physiologic levels of water loss. Changes induced by other solutions were not significant. Heterogeneity and variability of hydration-induced change in vocal fold tissues and functional voice measures is a common challenge in *ex vivo*, *in vivo*, and clinical studies [3–6]. For example, in the study by Oleson et al. [27], some rat vocal folds and salivary glands actually increased in intensity post dehydration. Similarly, in a clinical study, 3 out of 16 patients had increased vocal fold thickness on endoscopy after hemodialysis despite weight loss [51]. Our findings thus indicate that a higher number of larynges should be included in future work. There were no significant post-immersion intensity changes in anterior or posterior mucosa (Fig 4A and 4C). Due to the relatively steep angle of the porcine vocal fold within the thyroid cartilage [28,52–54] and 4-mm thickness of our slices, it is possible that selected points within anterior and posterior vocal folds included other tissue types (e.g. cartilage or fat) that were not the focus of this study. By contrast, the middle third of the mucosa was relatively isolated and measurements were more likely to include only mucosa or muscle. Taken together, these results demonstrate that response of vocal fold mucosa to solutions of varying tonicity is highly variable, and that 30-minute immersion in low-solute concentrations produces excessively high dehydration levels in the middle third of the mucosa.

There were no significant post-immersion intensity changes in muscle (Fig 4D and 4F), possibly due to limited depth of solution penetration for the duration of immersion in this study. Using hypertonic solutions for longer durations to dehydrate thyroarytenoid muscle is ill-advised, as this is the basis of brining meat, which results in increased moisture. Increased NaCl concentration below 5 molarity (M), roughly 29%, causes myofibril swelling, which is maximized at 1 M (5.8%) [55]. Therefore hypertonic immersion has little relevance to the effects of dehydration on muscle *in vivo*. Intensity change in mucosa after immersion in hypo-, iso-, and hypertonic solutions was not associated with change in muscle at the same anterior-posterior location (Fig 5A), which provides further evidence that this method can be used to analyze hydration-induced changes in these tissues separately.

After rehydration, vocal folds dehydrated in different hypertonic solutions or dry air did not differ in intensity in mucosa or muscle (S3 File). However, we found a positive association between intensity after dehydration and after rehydration in both mucosa and muscle (Fig 5C and 5D), indicating that the degree of change in water content due to immersion in hypertonic solutions persisted even after rehydration. Karamzadeh et al. [41] found that after dehydrating rabbit subglottis in 5% NaCl for 25 minutes, immersion for the same duration in H2O increased subglottic lamina propria from its dehydrated thickness. They did not quantify changes, so it is unclear whether tissue returned to baseline. Hanson et al. [33] dehydrated excised canine vocal fold mucosa by 30% and 70% of mass using a vacuum oven. Using 0.9% saline, they were able to restore mass of only half of tissues dehydrated by 30% and 1 of 10 dehydrated by 70%. Chan and Tayama [56] immersed canine vocal fold mucosa in a 25% sucrose solution for 25 min and rehydrated in H2O for 30 minutes. Rheology revealed that dehydration increased stiffness and viscosity, which was only partially recovered after rehydration. Consistent with these studies, our results suggest that *ex vivo* dehydration methods induce irreversible changes in vocal fold mucosa, which limits generalization to clinical settings.

This study has several limitations. Laryngeal position within the scanner may have influenced fluid content within vocal fold locations and limited our ability to draw conclusions based on anatomical variation. It is possible that moisture could have evaporated from the surfaces of the vocal folds during scanning, but applying a barrier to prevent this would have concentrated surface liquid in place by capillary action and confounded our measures. A single rater completed all measurements; however, intrarater reliability was excellent (ICC > 0.95). Mucosa was difficult to distinguish in dehydrated larynges, potentially resulting in imprecise measurements. Our dehydration method could not produce clinically applicable change in muscle [57] and produced excessively high dehydration of mucosa.

Water deprivation has different effects on cellular and extracellular fluid compartments of the body [58]. For example, ventricular volume in the brain paradoxically increases after acute dehydration, possibly due to diffusion of water out of cells and into extracellular space [35,36,38]. Thyroarytenoid muscle is largely cellular, and vocal fold mucosa comprises thick, largely acellular lamina propria and relatively thin cellular epithelium [12]. Due to the different effects of these tissues on phonation, it is important to understand hydration-induced changes in vocal fold mucosa and muscle as separate entities. We have demonstrated that PD-MRI can distinguish separate vocal fold tissue layers in a large mammalian larynx. Future studies should (1) focus on the middle third of mucosa, which has clinical relevance in biomechanical load and lesion occurrence [44–47], (2) include a higher number of larynges per condition, and (3) use shorter duration of immersion to produce less extreme changes in water content. *In vivo* studies are needed to quantify the relationship between change in vocal fold tissue and overall systemic hydration level using multiple measurements such as weight change, urine specific gravity, and serum osmolality [58].

## Conclusions

PD-MRI can be used to visualize porcine vocal fold tissue layers and to quantify changes in water content within vocal fold mucosa and thyroarytenoid muscle. Hypertonic solutions dehydrate the middle third of vocal fold mucosa but produce excessively high water loss in 30 minutes. Degree of change in vocal fold water content induced by hypertonic solutions *ex vivo* persists after rehydration.

## Supporting information

S1 File Post hoc testing within location × group interaction in mucosa intensity at baseline. (PDF)

S2 File Intensity differences from baseline after immersion. (PDF)

S3 File Intensity differences from baseline after rehydration. (PDF)

S4 File Raw data. Signal intensity of vocal fold tissues and phantom across baseline, immersion, and rehydration. Slices 1–80: experimental data. Slices 81–96: repeated measurements used to calculate intrarater reliability.
(XLSX)
