# Supplementary material for: Magnetic resonance imaging quantification of dehydration and rehydration in vocal fold tissue layers
Source: PLoS One. 2018 Dec 6;13(12):e0208763. doi: 10.1371/journal.pone.0208763 (PMC6283588; doi:10.1371/journal.pone.0208763)
Supplement: S1 File — (PDF) [file pone.0208763.s001.pdf]

S1 File. Post hoc testing within location × group interaction in mucosa intensity at baseline.

Table A. Effect of location in vocal fold mucosa within groups at baseline.

**Group**	**Result**	**Significance (p)**
H2O	F(2,96) = 2.01	0.1391
PBS	F(2,96) = 2.59	0.0805
Dry	F(2,96) = 3.43	0.0365*
5% NaCl	F(2,96) = 5.01	0.0085**
10% NaCl	F(2,96) = 0.46	0.6314
30% NaCl	F(2,96) = 18.14	<.0001***

Type 3 tests of fixed effects of location on vocal fold mucosa intensity within experimental groups in mixed model with intercepts for each larynx included as repeated measures. n = 5 larynges per group. *p<.05, **p<.01, ***p<.001.

Table B. Effect of group within location in vocal fold mucosa at baseline.

**Location**	**Result**	**Significance (p)**
Anterior	F(5,96) = 1.64	0.1566
Middle	F(5,96) = 2.87	0.0186*
Posterior	F(5,96) = 1.63	0.1592

Type 3 tests of fixed effects of group on vocal fold mucosa intensity within locations in mixed model with intercepts for each larynx included as repeated measures. n = 30 larynges. *p<.05.

Table C. Pairwise group comparisons within middle vocal fold mucosa at baseline.

	**PBS**	**Dry**	**5% NaCl**	**10% NaCl**	**30% NaCl**
**H2O**	0.9977	1.0000	1.0000	0.9942	0.7982
**PBS**		0.9951	0.8318	0.3895	0.0797
**Dry**			1.0000	0.9972	0.8464
**5% NaCl**				1.0000	0.9961
**10% NaCl**					1.0000

P-values for Tukey’s honestly significant difference pairwise tests of the hypothesis that there is a difference in intensity between groups. n = 5 larynges per group. All adjusted p > 0.05.
