# Supplementary material for: Magnetic resonance imaging quantification of dehydration and rehydration in vocal fold tissue layers
Source: PLoS One. 2018 Dec 6;13(12):e0208763. doi: 10.1371/journal.pone.0208763 (PMC6283588; doi:10.1371/journal.pone.0208763)
Supplement: S2 File — (PDF) [file pone.0208763.s002.pdf]

S2 File. Intensity differences from baseline after immersion.

Table A. Group differences in intensity within tissue and location after immersion.

Tissue and location	Result	Significance (p)
Mucosa		
Anterior	F(5,24) = 1.16	0.3554
Middle	F(5,10.8726) = 8.55	0.0017**
Posterior	F(5,24) = 1.32	0.2909
Thyroarytenoid		
Anterior	F(5,24) = 1.03	0.4242
Middle	F(5,24) = 0.30	0.9097
Posterior	F(5,24) = 0.65	0.6615

Results of one-way ANOVA and Welch's ANOVA testing the hypothesis that immersion in different solutions produces differences in intensity (% of baseline). n = 30 larynges. **p<.01.

Table B. Mean intensity by tissue, location, and group after immersion.

Location and group	Intensity (% of baseline) Mean	95% CI	Significance (p)
Anterior mucosa			
H2O	100.08	56.95-143.21	0.9960
PBS	112.41	71.50-153.31	0.4471
Dry	98.37	80.30-116.45	0.8149
5% NaCl	81.09	56.35-105.83	0.1011
10% NaCl	89.42	45.06-133.78	0.5441
30% NaCl	125.31	65.59-185.03	0.3045
Posterior mucosa			
H2O	106.41	91.01-121.81	0.3119
PBS	97.62	83.95-111.29	0.6536
Dry	99.61	76.14-123.08	0.9655
5% NaCl	94.23	49.31-139.14	0.7392
10% NaCl	104.63	75.23-134.04	0.6844
30% NaCl	73.94	42.56-105.32	0.0824
Anterior muscle			
H2O	101.38	76.59-126.16	0.8849
PBS	94.67	72.40-116.94	0.5425
Dry	114.45	66.88-162.02	0.4465
5% NaCl	88.54	75.54-101.54	0.0706
10% NaCl	93.54	74.36-112.72	0.4025
30% NaCl	90.20	75.27-105.13	0.1424
Middle muscle			
H2O	101.57	88.14-114.99	0.7624
PBS	99.86	84.34-115.39	0.9818
Dry	103.06	73.82-132.30	0.7857
5% NaCl	97.41	81.97-112.85	0.6654
10% NaCl	95.04	85.97-104.12	0.2038
30% NaCl	94.29	75.26-113.32	0.4519
Posterior muscle			
H2O	101.13	90.21-112.05	0.7880
PBS	97.37	85.79-108.97	0.5634
Dry	96.52	78.54-114.50	0.6193
5% NaCl	89.70	76.09-103.31	0.1035
10% NaCl	99.97	83.36-116.58	0.9962
30% NaCl	102.43	81.40-123.45	0.7648

Results of Welch's T-test of the hypothesis that intensity after immersion was not equal to 100% of baseline. n = 5 larynges per group. All p > Bonferroni-adjusted $\alpha = 0.05/6 = 0.0083$.
