# Supplementary material for: Magnetic resonance imaging quantification of dehydration and rehydration in vocal fold tissue layers
Source: PLoS One. 2018 Dec 6;13(12):e0208763. doi: 10.1371/journal.pone.0208763 (PMC6283588; doi:10.1371/journal.pone.0208763)
Supplement: S3 File — (PDF) [file pone.0208763.s003.pdf]

S3 File. Intensity differences from baseline after rehydration.

Table A. Group differences in intensity within tissue and location after rehydration.

Tissue and location	Result	Significance (p)
Mucosa		
Anterior	F(3,16) = 1.63	0.2215
Middle	F(3,16) = 0.83	0.4969
Posterior	F(3,16) = 2.31	0.1150
Thyroarytenoid		
Anterior	F(3,8.3910) = 0.21	0.8901
Middle	F(3,8.5719) = 0.34	0.7955
Posterior	F(3,16) = 1.56	0.2368

Results of one-way ANOVA and Welch's ANOVA testing the hypothesis that rehydration after immersion in hypertonic solutions and dry air produces differences in intensity (% of baseline). n = 20 larynges. All p > 0.05.

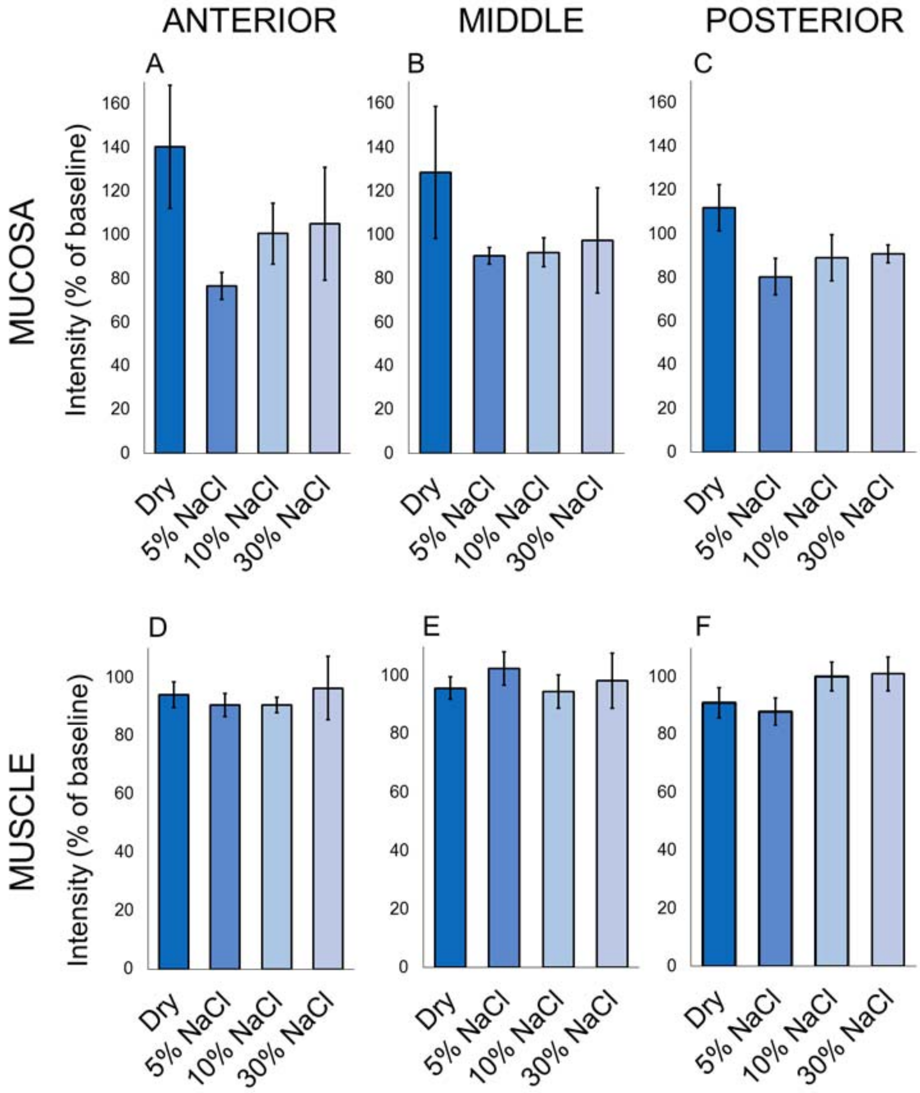

Fig. Intensity in vocal fold mucosa and thyroarytenoid after rehydration. Intensity (% of baseline) as a function of immersion solution in mucosa (A-C) and thyroarytenoid muscle (D-F). Data plotted as mean ± SEM. n = 5 larynges per group.

Table B. Mean intensity by tissue, location, and group after rehydration.

Location and group	Intensity (% of baseline)		Significance (p)
	**Mean**	**95% CI**	
Anterior mucosa			
Dry	140.32	62.15-218.49	0.2254
5% NaCl	76.67	59.73-93.62	0.0187
10% NaCl	100.66	62.10-139.21	0.9646
30% NaCl	105.07	33.43-176.70	0.8539
Middle mucosa			
Dry	128.52	44.60-212.43	0.3988
5% NaCl	90.35	79.55-101.16	0.0683
10% NaCl	91.94	73.78-110.10	0.2853
30% NaCl	97.48	31.07-163.89	0.9213
Posterior mucosa			
Dry	111.99	82.56-141.43	0.3211
5% NaCl	80.43	57.47-103.38	0.0770
10% NaCl	89.05	59.53-118.57	0.3614
30% NaCl	90.86	79.40-102.33	0.0913
Anterior muscle			
Dry	93.95	81.84-106.07	0.2381
5% NaCl	90.53	79.44-101.61	0.0766
10% NaCl	90.48	83.16-97.80	0.0225
30% NaCl	96.27	66.11-126.44	0.7488
Middle muscle			
Dry	140.32	62.15-218.49	0.2254
5% NaCl	76.67	59.73-93.62	0.0187
10% NaCl	100.66	62.10-139.21	0.9646
30% NaCl	105.07	33.43-176.70	0.8539
Posterior muscle			
Dry	91.18	76.66-105.69	0.1668
5% NaCl	88.04	74.91-101.17	0.0647
10% NaCl	100.25	86.37-114.14	0.9621
30% NaCl	101.14	84.81-117.46	0.8558

Results of Welch's T-test of the hypothesis that intensity after rehydration was not equal to 100% of baseline. n = 5 larynges per group. All $p > $ Bonferroni-adjusted $\alpha = 0.05/4 = 0.0125$.
